# Post-COVID-19 rehabilitation: a special look at chronic kidney disease patients

**DOI:** 10.1186/s41100-021-00355-7

**Published:** 2021-06-15

**Authors:** Heitor S. Ribeiro, Amanda E. Rodrigues, Jennifer Cantuária, Antônio Inda-Filho, Paul N. Bennett

**Affiliations:** 1 Faculty of Physical Education, University of Brasília, Campus Universitário Darcy Ribeiro, Brasília, DF ZIP code 70910-900 Brazil; 2 Research Center in Sports Sciences, Health Sciences and Human Development (CIDESD), University Institute of Maia (ISMAI), Porto, Portugal; 3 University Center ICESP, Brasília, Brazil; 4 Department of Health Sciences, Federal University of Goiás, Goiânia, Brazil; 5 RenalCare Rehabilitation Center, Goiânia, Brazil; 6 Satellite Healthcare, Inc., San Jose, CA USA; 7 University of South Australia, Adelaide, Australia

**Keywords:** Exercise, Rehabilitation, Kidney replacement therapy, SARS-CoV-2, Preventive medicine

## Abstract

**Background:**

Severe acute respiratory syndrome coronavirus 2 (SARS-CoV-2) can infect the kidney and the presence of chronic kidney disease (CKD) constitutes a higher risk of negative prognosis. SARS-CoV-2 main sequelae in CKD patients are an incomplete recovery of kidney function, muscle weakness and atrophy, breathiness, tiredness, pulmonary fibrosis, and initiation of kidney replacement therapy. The overall aim of this review is to provide a theoretical basis for early improvements of physical function health to all CKD stages by rehabilitation therapies.

**Conclusion:**

Chronic kidney disease patients infected with SARS-CoV-2 should be monitored by rehabilitation professionals as the cardiopulmonary, musculoskeletal, and cognitive systems might be deteriorated. Long-term consequences of SARS-CoV-2 are unknown and preventive rehabilitation may attenuate them.

## Kidney as a target of SARS-CoV-2

Severe acute respiratory syndrome coronavirus 2 (SARS-CoV-2) can infect the kidney [33]; however, it is not yet clear if virus replication occurs resulting in functional damage. Given the expression of the angiotensin-converting enzyme 2, the kidney is vulnerable to SARS-CoV-2. If kidney dysfunction is caused only by direct damage of the virus or is secondary also to other systemic processes triggered by SARS-CoV-2 it has not been well described. In a cohort of 701 SARS-CoV-2 chronic kidney disease (CKD) patients, the presence of proteinuria and hematuria were associated with an increased risk of in-hospital death up to 11- and 12-fold, respectively [9]. Also, a systematic review and meta-analysis showed that CKD patients were more likely to be transferred to intensive care and undergo mechanical ventilation [17]. Therefore, the presence of CKD on admission constitutes a higher risk of a negative prognosis [5].

## Main sequelae expected in CKD patients

Kidney manifestations have been frequently associated with SARS-CoV-2 and unique characteristics in individuals with previous CKD [13]. The prevalence of pre-existing CKD is an independent risk factor for acute kidney injury and, followed by hyperkalemia, is the most common kidney complication in the context of coronavirus disease (COVID-19) [12, 24, 43]. As expected, due to its persistent pro-inflammatory state and its functional defects in innate and adaptive immunity, CKD increases the chances of infection in these patients, as well as their development in the most severe forms, and can lead to death [17, 21]. Rhabdomyolysis and metabolic acidosis are also common and are almost always associated with hemodynamic instability [32].

**Fig. 1:**
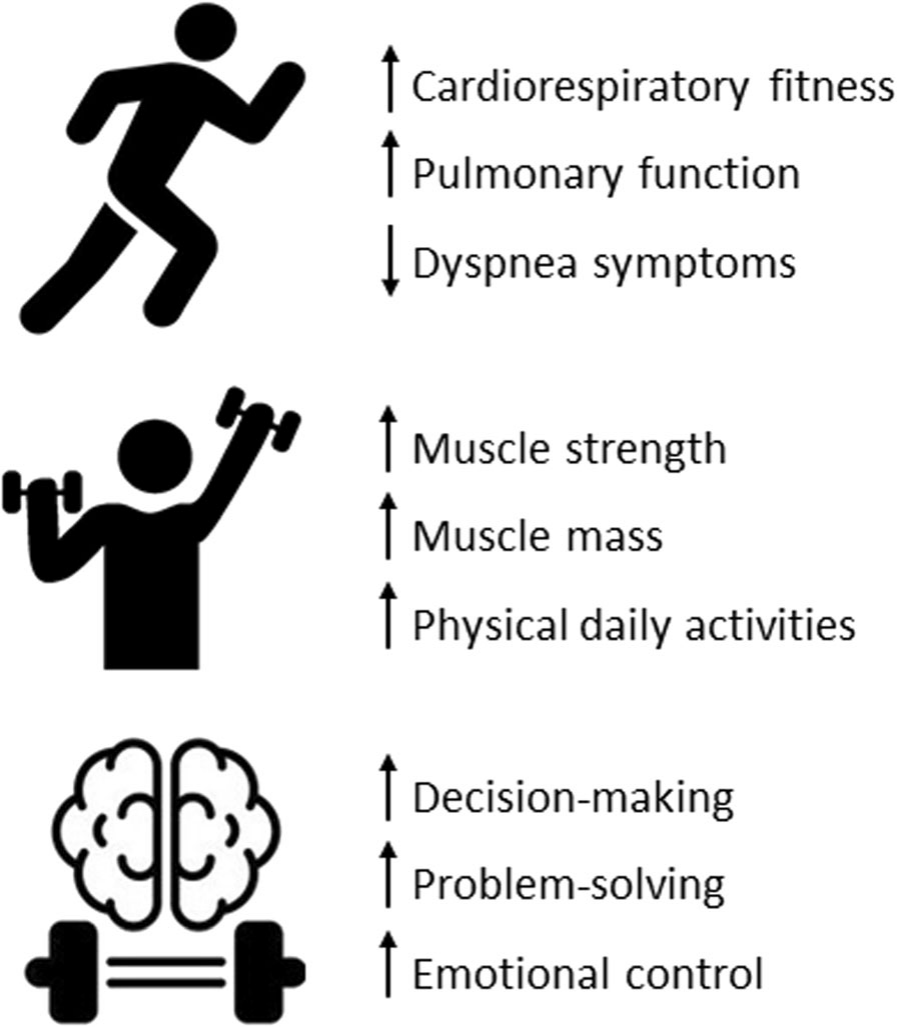
Rehabilitation therapies benefits

Regardless of whether kidney function is altered on admission or developed during hospitalization, many people experience kidney function loss after hospital discharge [29]. A significant number requires long-term follow-up due to incomplete recovery of kidney function, continuous interstitial inflammation, loss of renal vascular cell regenerative potential, and hypertension [21]. The high cost of kidney replacement therapies and the lack of uniform availability of hemodialysis clinics manifest into a challenging scenario. Improving the outcome of these patients is fundamental and emerging; these sequelae cannot become a COVID-19 legacy. For this reason, exercise rehabilitation therapies may play an important role in improving physical function health and attenuating the expected sequels in CKD patients infected by SARS-CoV-2, as seen in Fig. 1.

## Cardiopulmonary rehabilitation

Kidney damage during SARS-CoV-2 infection is a risk factor for CKD development. The COVID-19 survivors, especially those dialysis-dependent or with pre-existing CKD, need to be closely monitored, as they represent a high-risk group [39]. Studies suggest that pulmonary fibrosis will become one of the main sequelae in patients with SARS-CoV-2 infection [6], which may be exacerbated in CKD. Lung damage associated with SARS-CoV-2 can lead to the impairment of alveolar air exchange and a decrease of pulmonary ventilation function [25]. As a result, many patients reported respiratory symptoms such as dyspnea and chest tightness, and almost half within 1 month after SARS-CoV-2 infection have developed pulmonary fibrosis, persisting up to 6 months. During hospitalization, the development of cardiac complications such as acute myocardial injury, arrhythmias, and cardiogenic shock may also be seen, increasing mortality risk in AKI and CKD survivors.

It is known that CKD is associated with the concomitant development of cardiopulmonary diseases, resulting in poor cardiorespiratory fitness and all its deleterious consequences [27]. Lifestyle changes, medication adequacy, health education, and a rehabilitation program with therapeutic exercises can alleviate kidney damage and improve patient outcomes in the long term [2]. The aim of rehabilitation in the context of cardiopulmonary complications of SARS-CoV-2 is to trigger the systemic antioxidant response to modulate the inflammatory state generated by the virus and to intervene in the endothelial dysfunction caused by it. This can be achieved through exercise rehabilitation, among which the most used types are: aerobic, respiratory, resistance, and interval training [7].

Patients with SARS-CoV-2 sequelae are almost always characterized by respiratory problems of varying degrees; for this reason, a cardiopulmonary rehabilitation protocol must be applied and customized based on the specific sequelae of each individual; in this context, the respiratory muscle training has been used as an important tool in the recovery of these patients [11]. Therefore, it is necessary to have an in-depth knowledge of the probable and serious sequelae that surviving CKD patients may develop, as well as the development of action plans to deal with this situation, from the discharge process to the insertion in cardiopulmonary rehabilitation programs.

## Musculoskeletal rehabilitation

SARS-CoV-2 infection can require bed-rest due to fatigue, dyspnea, tiredness, and breathlessness. Moreover, those who needed to be admitted to intensive care units may have been hospitalized for up to 21 days [22]. Long hospitalization periods are associated with muscle dystrophy, systemic inflammation, and musculoskeletal atrophy [4]. It is widely known that CKD patients commonly experience muscle-wasting, muscle weakness, and impaired physical function [35]. Thus, CKD patients affected by SARS-CoV-2 may be at high risk for musculoskeletal health impairments, such as sarcopenia, dynapenia, protein-energy wasting, cachexia, and frailty [34]. Practical approaches to mitigate these possible adverse effects of SARS-CoV-2 in CKD patients’ musculoskeletal health should be, therefore, a high priority.

Physical function rehabilitation performed with resistance training, balance exercises, and neuromuscular electrical stimulation could potentially counterbalance muscle and strength losses due to muscle disuse caused by bed-rest and long hospitalization periods [28]. We, therefore, recommend rehabilitation professionals start early interventions in the acute inpatient setting, such as passive mobilization, bed mobility, sit-to-stand, and isometric exercises, and for safety reasons, control all clinical parameters [38]. When it comes to hospital discharge, CKD patients infected by SARS-CoV-2 should be continued into physical rehabilitation. Home-based, in-home telehealth, intradialytic, or patient-directed exercises determined to patient needs should be delivered [3, 14].

Additionally, supplementation with high-quality protein has been shown to improve physical function and inflammation in CKD patients [41], thus, combined with exercise may play an important role in preserving musculoskeletal health. Considering nutrition and diet holistic aspects of COVID-19 infection, previous studies have already elucidated its importance [18, 31].

## Cognitive rehabilitation

The vital interplay between psychological and physical health requires the consideration of cognitive rehabilitation to enhance cardiopulmonary and musculoskeletal rehabilitation. In addition to the somatic symptoms of SARS-CoV-2, quarantine and minimal contact with family and friends can increase fear, stress, and anxiety. This can lead to cognitive deficits related to decision-making, problem-solving, memory, attention, and emotional control [44]. Evidence is emerging highlighting the deficiency in the cognition of SARS-CoV-2 survivors as measured by validated psychological tests such as the Continuous Performance Test [45].

Acknowledging and assessing debilitative cognitive dysfunction in CKD patients is the first step. If cognitive dysfunction is present, cognitive rehabilitation therapy (CRT) may be considered to improve functioning to decrease the debilitating effects of cognitive decline. CRT describes approaches that can restore and enhance cognitive performance and can provide strategies (e.g., mnemonics, memory notebooks) to assist a person with activities of daily living in the presence of cognitive dysfunction [23]. CRT could be performed via telehealth, particularly if face-to-face SARS-CoV-2 restrictions existed [36].

Table 1 summarizes the exercise rehabilitation recommendations for CKD patients for cardiopulmonary, musculoskeletal, and cognitive systems [1, 15, 16].

**Table 1:** Post-COVID-19 rehabilitation recommendations for chronic kidney disease patients

	Modality	Volume	Intensity	Frequency
**Cardiopulmonary**	Aerobic exercises	Up to 150 min/week	Low to moderate intensity (50–70% HRmax)	3 weekly sessions
	Breathing exercises	3–5 sets of 10 repetitions/day	30% of peak inspiratory pressure	5 weekly sessions
**Musculoskeletal**	Resistance exercises	8–10 sets of 12–15 repetitions/week	4–7 at OMNI-RES scale; 50–70% 1RM	2 weekly sessions
**Cognitive**	Cognitive Rehabilitation Therapy	15 to 30 min	Depending on cognitive deficit	Daily CRT with weekly professional support via telehealth

*HR* heart rate, *CRT* cognitive rehabilitation therapy, *OMNI-RES* OMNI resistance training scale, *1RM* one repetition maximum

## Rehabilitation nuances for CKD and general population

Most of the recent evidence regarding rehabilitation programs for post-COVID-19 survivors is coming from the general population and there is a lack of evidence for CKD, as well as other pre-existing chronic diseases [8, 10, 26]. Daynes et al. [10] showed that a 6-week, twice-supervised rehabilitation program, consisted of aerobic exercise, strength training, and educational discussions improved fatigue, breathlessness, exercise capacity, and cognition. Previously, Liu et al. [26] demonstrated that a 6-week respiratory rehabilitation program was able to improve respiratory function, quality of life, and anxiety in the elderly. Both studies showed no adverse events related to the rehabilitation program and appeared to be safe and effective in improving physical health in general post-COVID-19 survivors.

Based on the limited evidence with the general population, we speculate that the same benefits would be found in CKD patients. Previous studies have shown that CKD patients have worse cardiopulmonary [30], musculoskeletal [37], and cognitive [42] functions than non-CKD subjects. Post-COVID-19 survivors with CKD may have experienced an even worse impact on their physical health than non-CKD. Thus, rehabilitation programs should also be addressed to CKD patients in an attempt to attenuate COVID-19-related sequelae, as previous studies have shown it to be safe and effective in non-CKD. However, there are some specificities related to CKD that need to be addressed and preferable before the commencement of a rehabilitation program in these patients:

- Kt/V > 1.2 (dialysis patients) [20]
- Inter-dialytic weight gain > 4 kg should be avoided [40]
- Hemoglobin concentration ≥ 13.0 g/dL in males and ≥ 12.0 g/dL in females [19]
- Monitor for electrolyte abnormalities (i.e., hypo/hyperkalemia, hypo/hypercalcemia, hypo/hypermagnesemia, hypo/hyperphosphatemia) [40]
- Evaluate for pulmonary congestion, pleural effusion, pulmonary hypertension, and/or peripheral edema [40]

## Conclusions

Chronic kidney disease patients affected with SARS-CoV-2 should be monitored by rehabilitation professionals as the cardiopulmonary, musculoskeletal, and cognitive systems might be deteriorated due to the infection. During the infection phase, if the patient is physically able to rehab (i.e., not reporting fever or dyspnea, oxygen saturation ≥ 95%, rhythmic heart rate), it should be started respecting all safety procedures to avoid the therapist’s infection, but home-based or telehealth sessions should be prioritized. Long-term consequences of SARS-CoV-2 on physical function health are unknown and preventive rehabilitation may attenuate them. Therefore, future experimental studies must be designed to elucidate the rehabilitation benefits of SARS-CoV-2-related sequelae in CKD patients from all stages.

## Data Availability

Not applicable.
